# Balancing health care evidence and art to meet clinical needs: policymakers' perspectives

**DOI:** 10.1111/j.1365-2753.2009.01209.x

**Published:** 2009-12

**Authors:** Louise E Parker, Mona J Ritchie, JoAnn E Kirchner, Richard R Owen

**Affiliations:** 1 Independent Researcher and Consultant, Cambridge, MA, USA, Consultant, HSR&D Center for Mental Healthcare and Outcomes ResearchNorth Little Rock, AR, USA and Consultant, Mental Health Quality Enhancement Research InitiativeNorth Little Rock, AR, USA; 2 Investigator, HSR&D Center for Mental Healthcare and Outcomes ResearchNorth Little Rock, AR, USAAdministrative Coordinator, Mental Health Quality Enhancement Research InitiativeNorth Little Rock, AR, USA and Project Director, University of Arkansas for Medical SciencesLittle Rock, AR, USA; 3 Associate Director for Clinical Care, VA South Central Mental Illness Research, Education, and Clinical CenterNorth Little Rock, AR, USA, Associate Professor, University of Arkansas for Medical SciencesLittle Rock, AR, USA and Investigator, HSR&D Center for Mental Healthcare and Outcomes ResearchNorth Little Rock, AR, USA; 4 Center Principal Investigator, HSR&D Center for Mental Healthcare and Outcomes ResearchNorth Little Rock, AR, USAResearch Coordinator, Mental Health Quality Enhancement Research InitiativeNorth Little Rock, AR, USA and Professor, University of Arkansas for Medical SciencesLittle Rock, AR, USA

**Keywords:** art of medicine, clinical need, evidence-based health care, evidence-based medicine, evidence-based practice

## Abstract

**Rationale, aims and objectives:**

Although many believe that evidence-based practice (EBP) has great potential, critics have identified limitations including a focus on randomized clinical trial (RCT) evidence to the exclusion of other evidence types and a disregard for the art of medicine. Others have argued, however, that proper application of EBP involves reasoned consideration of a wide variety of information; thus, the dichotomy between medical science and art may be false. We explore the views of executive-level policymakers from the Veterans Health Administration, a leader in the EBP movement, regarding what constitutes evidence and the relative importance of evidence versus practical needs when determining clinical policy.

**Method:**

We conducted 26 semi-structured qualitative interviews and performed a content analysis.

**Results:**

Although informants generally believed in the value of EBP and the role of RCTs within it, they also valued other types of evidence. Further, they had concerns that were sometimes antithetical with strict adherence to an evidence-based approach. These included practical concerns, fit with organizational values and with local circumstances, resources, political pressures and patient needs. They were especially concerned about how to address medical conditions that affect many individuals or high-risk populations that have no evidence-based treatment.

**Conclusion:**

When possible, health care practice should be evidence-based. When this is not possible, health care providers must turn to the art of medicine by using consensus-based best practices. Further, it is important for policymakers and researchers to work in concert to develop EBPs that are practical and meet needs.

## Introduction

Historically, health care practitioners have considered their work as much an art as a science, drawing upon clinical intuition honed by years of experience [1]. Since the early 1990s, however, health care practice based on scientific empirical evidence, known variously as evidence-based medicine, evidence-based health care, and most generically, evidence-based practice (EBP), has taken the health care industry by storm [2–4]. Although many health care researchers, policymakers, and managers believe that basing health care practices upon empirical evidence has great potential to improve patient outcomes, critics have identified a number of limitations [5]. Further and somewhat ironically, there is no clear evidence that EBP produces superior care and outcomes [6–8].

Much of the concern focuses on the assumption among many EBP proponents that randomized clinical trials (RCTs), especially double-blind trials, should provide the gold standard for clinical evidence. Critics of this stance have argued that typical patients are often more complicated and less compliant than those included in RCTs [7,9–14]. Moreover, what we observe on average in groups may not apply to a particular individual or context; by contrast, the art of medicine accommodates individual patients', providers', health care organizations', communities' and societies' needs, preferences and circumstances [4,7,12,14–17]. Additionally, as RCTs' focus is the effect of treatments on final outcomes (e.g. the number of patients who experience less of a particular symptom), they do not explore the complex biological processes underlying disease and health [15,18].

Interestingly, despite many EBP advocates' claims that RCTs provide the highest quality evidence, findings from observational studies actually converge more often than do findings from RCTs [6]. Further, health care evidence is a ‘fuzzy set’ in that there are not always clear boundaries between what is and what is not effective across studies [19]. Thus, it seems imprudent to assume that one type of methodology provides the only path to knowledge. We should note that many of the researchers and policy analysts who are concerned about the centrality of RCT within the EBP movement are not claiming that RCTs are valueless. Rather, these critics propose that clinical decision makers should make reasoned use of all available information, including qualitative and correlation studies, providers' clinical observations and context, patients' experiences and context, as well as RCTs [3,8,16,20,21].

A number of health services researchers have observed that qualitative methods (e.g. case studies, qualitative interviews and qualitative observational studies) provide particularly viable approaches for exploring the needs and preferences of health consumers who do not fit neatly into the types of groups RCTs require. These include those with co-morbid conditions or complex life circumstances [9,22,23,24]. Additionally, before researchers can test treatments in an RCT, someone needs to discover them. Many researchers reason that qualitative methods, such as case studies, may play an important role in treatment discovery [6,15,16,25]. Qualitative methods are also valuable in discovering new diseases and in describing disease mechanisms and treatment side effects [18].

There are also quantitative methods other than RCT that can provide valuable information regarding disease process, treatment, and prevention. Although only experimental methods, such as RCT, can establish causal relationships, quantitative correlational methods are also valuable tools, especially where experiments may be unethical. For example, physicians generally recommend that patients do not smoke based on the well-established correlation between smoking and lung disease rather than on any experiment definitively demonstrating that smoking causes such disease. Finally, Buetow and Kenealy proposed that EBP should include not just research evidence, whether it is qualitative or quantitative, but also theoretical, practical, experiential, expert, judicial and ethical evidence [26].

Also of concern is the nature of research publication and funding. As journal editors prefer to publish statistically significant positive results, reviews of the research literature may over estimate true effect sizes. This issue, known as the ‘file draw’ problem or effect because negative findings do not enter the literature but rather literally languish in investigators' file draws, affects all sciences. Quite simply, it is far easier to learn of trials where treatments do work than it is to learn of those where they do not. Further, there is more research funding for commercially viable interventions (e.g. profitable pharmaceuticals) than for interventions with limited commercial value (e.g. lifestyle and dietary changes). Additionally, some treatments (e.g. pharmaceuticals) are more amenable to EBP methods, such as double-blind RCT, than others (e.g. lifestyle changes, psychotherapy and many procedures). Thus, there may be more published evidence created for certain types of treatments than for others, regardless of their actual value [2,4,7,11]. Finally, EBP sits at the nexus of the current conflict between a traditional perhaps idealized view of health care in which practitioners provide the best care, regardless of circumstances and costs, and a ‘rationalized’ system of care [11].

On the other hand, numerous health services researchers and policy analysts have argued that proper application of EBP does not involve dogmatic adherence to RCT results alone. Rather, EBP should include consideration of evidence from a wide variety of sources, as well as individual patient and provider experiences and local context [1,3,6,20,21,25,27,28]. As such, the dichotomy between EBP and medical art may be false.

In this article, we explore the views of US Veterans Health Administration (VHA) executive-level policymakers regarding the relative importance of research evidence and of patient and other needs when determining clinical policy. We also explore their views on what they believe constitutes evidence. We believe the VHA provides a particularly important context for exploring these issues because it is one of the world leaders in the development and implementation of EBP. Most notably, the VHA established the Quality Enhancement Research Initiative to foster the translation of research evidence into clinical care [29]. Further, VHA medical centres are academically affiliated and medical schools have largely embraced EBP [12].

## Methods

### Data collection

As part of a study concerned with understanding how to implement and sustain organization-wide clinical quality improvement (QI) programmes, we identified a purposive sample of 28 current and former executive-level VHA policymakers and key members of their staff. Twenty-six of these agreed to participate in the study and we were able to conduct 12 of these interviews face-to-face at informants' offices during two site visits to the VA Central Office in Washington, District of Columbia, USA. We interviewed 14 informants who were unavailable for face to face interviews or who had offices in locations other than Washington via telephone.

Three highly experienced qualitative interviewers, a psychologist, a psychiatrist and a social worker, conducted all the interviews. Two of these three interviewers participated in each interview with one serving as the lead interviewer and the other serving as a back-up interviewer taking notes and ensuring that the lead interviewer covered all crucial topics. The three interviewers took turns serving in these capacities.

We conducted all interviews utilizing a qualitative semi-structured protocol. Our protocol was semi-structured in that we developed a topic list but we did not ask exactly worded questions in an exact order and we did not have a closed response set. Qualitative methods are useful when there is limited a priori knowledge regarding the constructs of interest and the researchers seek to discover new information rather than confirm existing hypotheses. Topic areas for this study included fostering partnerships between VHA policymakers and researchers, integrating new clinical programmes into the VHA structure, and the balance between evidence and need in determining VHA policy. The latter topic is the focus of this article. We audio-recorded all interviews and produced verbatim transcripts.

### Data analysis

Utilizing Atlas.ti, a qualitative data management software package, we conducted a content analysis of the verbatim transcripts [30]. Specifically, we linked codes to passages within the transcripts (i.e. quotations). Content analysis, one of the most commonly used qualitative data analysis techniques, is similar to creating a book index [31]. Data analysis for this study proceeded over two phases.

First, two investigators reviewed the interview transcripts and inductively developed top level codes that described major themes. These included: (1) what convinces informants to support programmes; (2) how to integrate programmes into the VHA structure; (3) issues related to various VHA departments; (4) barriers to researchers partnering with clinical managers and VHA central office policymakers; and (5) the balance between evidence and clinical need in determining clinical policy. The two investigators then each coded one half of the transcripts and reviewed 100% of each other's coding and resolved any differences.

Second, working in teams of two, investigators reviewed all quotes linked to a particular top level code and inductively created subcategories or sub-codes that described the content of the quotes. Utilizing this sub-coding scheme, they each sub-coded one half of the transcripts. Finally, they reviewed 100% of each other's coding and resolved any differences.

In this article, we focus on the sub-codes for the ‘balancing evidence and need’ top level code. Sub-codes concern: (1) value of EBP in determining policy; (2) types of evidence valued; (3) limits to EBP and the role of the art of medicine when addressing concerns (e.g. fitting with local clinic circumstances, fitting with organizational and professional values, and meeting needs for which there may or may not be evidence-based treatments); (4) types of needs identified; (5) the ways in which clinical evidence may not meet existing needs; (6) methods for meeting needs in the absence of an applicable or complete evidence base; and (7) utilizing needs to guide the direction of research so as to collect useful evidence.

## Results

### EBP and types of evidence valued

All else being equal, our informants generally believed that basing clinical policy upon research evidence enhances quality. Many believed that RCT provides the highest standard evidence:

Well, obviously I think the level one evidence [RCTs] is the most compelling … [I]f they show us a research results that are from random control trials, those speak volumes …

At the same time, they understood that RCTs were not always viable:

As … someone … committed to the evidence-based psychotherapies … there's some things you have to push against, like the assumption that the gold standard is a randomized controlled double-blind trial. Because, obviously, you can't do psycho-social research … blind.

Further, they also valued other types of evidence including qualitative:

I want to see actually all of that [RCT, case studies, and testimonials]. And I value all of that. So I wouldn't just want to see it from just necessarily one approach. I don't think that addresses the whole issue.

[Case studies] play huge training roles … [R]eading the case studies can give you more of a feel for what's this like and what's the emotional experience of the patient, of the therapist, you know, what are the choice points you reach and how are those dealt with, how do you understand that approach to therapy in a richer way.

I think that the other evidence that I'm always interested in is [the new practices'] acceptability to the patient. And some of those things are sort of embedded in research reports but are perhaps not emphasized. So drop out rates, you know. Sometimes they're just treated as a source of kind of error but I think they're pretty profoundly meaningful.

### Beyond evidence: what else matters when making clinical policy?

#### Practicality

As much as they valued evidence, however, informants also believed there are other concerns when making policy decisions. First, clinical programmes have to be practical:

[T]he practicality of it is a big part … has it been tested in a typical environment or multiple typical environments … And I wouldn't say that we should always rely on … the rigor of the most pure science because then we would probably never get done because it is usually so narrow that it's hard to put your arms around it and say that it applies to multiple settings if it's been tested in one setting … [S]ince we're a provider organization, a practice environment, it has to work in the practice environment.

#### Fit with values and local circumstances and context

How well practices fit with local clinical and individual values, circumstances and context also factored into informants' decision making:

[T]he evidence is only one part of evidence-based care … other components … include understanding and incorporating … values, hopefully the patient's values, and the context … [T]his fellow is left-handed and has red hair and there's no one in the clinical trials who's left-handed and has red hair. So is the context such that the evidence can be applied to this person here and now or not? That's a matter of very fuzzy clinical judgment … [T]he values in Memphis may be different than … values in Cleveland, and that should affect what you do. And the context may be different as well and to fit between the hard evidence and the soft patient may vary so much that it begs the case … I think there's still more to clinical care than applying evidence only.

#### Resources

Informants also believed it is important to consider organizational resources:

There are great ideas that we could do and they could be very expensive and in the end the benefit is arguable, negligible.

I want to know that they've thought through the resources that it would take … [T]oo many times, you know, people want to do something, but they don't think through that you're adding it on top of already busy people, and this is something that is collateral duty now or is it something that's going to replace something else.

#### Political climate

Further, health care organizations, especially government funded ones such as VHA, must be cognizant of the political climate when making policies:

I think that we always remember where we work and who our various bosses are. And we have the White House [U.S. President] three blocks from here and then those folks at the east end of Pennsylvania Avenue [U.S. Congress]. And so a lot of what we end up doing as a government agency is driven by those particular dictates.

[W]hat's priorities for the groups that support us, Congress, [Veteran Service Organizations], et cetera. Sometimes you get initiatives that … from our perspective may not be the top impact clinically, but for other reasons … are strongly being … recommended.

#### Patient need

Finally, informants considered the needs of patients paramount in determining policy:

[I]f you go back to our vision statement for our system, it reads, to be a patient-centered system … I think all of these things should be characterized as we're pursuing this because this is an intervention that is needed by our population or at risk for these things.

I like to hear the volume of the patients that this will impact, and I also want to know if this is a high-risk population, because you can't just go by volumes. Sometimes you need to do it because it may be a small amount, but it's a high-risk population.

### In the absence of evidence, use consensus and work to build the evidence base

Given that informants believed that some needs are so compelling that it is important to attempt to meet them even in the absence of research evidence, it is necessary to identify other information to assist with decision making. For most, clinical consensus was the best approach in these instances:

[S]ometimes the highest level of evidence is consensus, and you have to understand that that may be all you get … If you wait for level-one evidence for everything, you would do hardly anything…. The NCCN [National Comprehensive Cancer Network] guidelines for cancer, for example, they'll tell you right up front they're consensus guidelines. But it doesn't mean you shouldn't do it just because they're consensus guidelines. That's the best thing we had, so that's what you use.

In such instances, however, informants would encourage proponents to establish an evidence base:

If the public health need is serious and urgent enough, I will go for less well-established evidence. But if I do that, then I would push that somebody ought to be parallel with the implementation actually work on establishing the evidence base. In other words, I will use so called best-practices, so often anecdotal but then I would say to the proponents that you need to at least show me that you are working on this to establish its effectiveness or efficacy.

To further the goal of increasing the percentage of EBP, many of our informants believed that clinical needs, rather than the interests of academics, should guide research, at least in organizations with clinical missions:

[W]e're looking much more carefully at veteran centricity and applicability of what we do across all the services … [Y]es, we want to attract this wonderful investigator who does basic science research [but] … If they want to do research, they have it's got to be much more hands on and veteran centric.

Ultimately, however, the informants saw the need for balancing the immediate needs of patients and the long-term scientific goals when setting research agendas:

I think it has to be interactive and sometimes the process should be driven by the needs of the programme or policy, and sometimes it should be driven by the scientific opportunities that are ready for development. You know, I don't speak either/or … But, gee, you know, I'm just I think we have to use the evidence as well as we can to meet the real needs. It would be silly, I think, to do otherwise.

Finally, policymakers argued that following a particular line of evidence blindly is unlikely to achieve sustainable QI. In other words, to remain faithful to the spirit of the QI initiative, health care organizations must listen and learn from the field, as well as the laboratory, and modify their practices accordingly:

So in essence, you know, the way to sustain is to actually change. The way to maintain fidelity is actually to change what it means to be faithful … because I think some of the things that may actually represent lack of faithfulness to the intervention may actually be responses to needs that the original model didn't fit … I think it's very simplistic to say … fidelity means doing things just like in that trial. I mean, you could pick apart anything … even the studies that have been done … [V]ery few studies actually get into the level of the ingredients of the intervention and … what inside the black box is truly needed and how much of each of those things … [W]e … know … it's nice to lower LDL cholesterol. We actually really don't know to what level we should lower LDL cholesterol and necessarily how we should lower it.

## Conclusions

It did not surprise us that these VHA executive-level policymakers were strong proponents of EBP. As we mentioned earlier, VHA is a leader in promoting EBPs. Further, and as we also indicated, VHA's nation-wide network of medical centres is affiliated with medical schools and academic medicine has embraced EBP. Interestingly, although these policymakers generally believed that RCTs are valuable, they also believed that other forms of evidence, including qualitative evidence, can be useful. Additionally, they observed that it is not possible to conduct RCTs for certain types of treatments.

The individuals that we interviewed, however, were also responsible for developing policy for and administering a large public health system. Thus, they had many concerns that were sometimes in conflict with strict adherence to an evidence-based approach. First, they believed that practices have to be practical in real world clinical settings. They also believed that practices have to fit with organizational values and with local circumstances. Further, they understood that as a government funded agency, VHA must respond to political pressures. These include addressing the needs of elected officials and the groups that lobby those officials. Finally and most importantly, they believed it is important to attempt to meet compelling patient needs. They were most concerned about conditions that affect large numbers of individuals or affect high-risk populations. When there was no clear evidence regarding how to address those needs, they believed health care organizations must turn to the art of medicine by using the collective wisdom of the field in the form of consensus-based best practices.

What these policymakers seemed to imply is that just as it would be foolish to ignore research evidence when adopting clinical policies, it would be equally foolish to ignore practical concerns, values and the needs of health care consumers. For serious conditions that have no clear EBP, practitioners must alleviate suffering by using the art of medicine in the form of best practices. Further, if individuals do not seem to respond to the usual treatment, then clinicians must try something else. And even when following the evidence, we must realize that we are always refining what we know. The best practice is an ever-evolving one that makes the best use of science and art to provide the best care possible to many varied individuals. It is also one that makes reasoned and critical use of all the available information. Lewis may have put it best when he wrote:

Where knowledge is definitive, rely on the algorithm; where knowledge is provisional and incomplete, redouble the scientific effort; where uncertainty looms large, engage the right side of the brain (p. 171) [7].

All of this implies that those concerned with building the evidence base and those concerned with delivering patient care must work in concert, designing interventions that are broadly applicable to actual clinical practice [32–34]. Research and practice, however, are strange bedfellows. Whereas research is a slow and deliberate process, the need to improve clinical care is often time-sensitive. Additionally, researchers have career demands (e.g. the need to develop a coherent research programme and become an expert in a particular area) that may not necessarily follow public health needs. Ultimately, however, the quality of health care depends on finding a way to blend medical art and science.

## References

[1] Greenhalgh T (2002). Intuition and evidence – uneasy bedfellows?. British Journal of General Practice.

[2] Gupta M (2003). A critical appraisal of evidence-based medicine: some ethical considerations. Journal of Evaluation in Clinical Practice.

[3] Jenicek M (2006). The hard art of soft science: evidence-based medicine, reasoned medicine or both?. Journal of Evaluation in Clinical Practice.

[4] Mykhalovskiy E, Weir L (2004). The problem of evidence-based medicine: directions for social science. Social Science Medicine.

[5] Ashcroft RE (2004). Current epistemological problems in evidence based medicine. Journal of Medical Ethics.

[6] Haynes RB (2002). What kind of evidence is it that evidence-based medicine advocates want health care providers and consumers to pay attention to?. BioMed Central Health Service Research.

[7] Lewis S (2007). Toward a general theory of indifference to research-based evidence. Journal of Health Services Research and Policy.

[8] Upshur RE (2006). Evidence-based medicine, reasoned medicine or both? Commentary on Jenicek, M. (2006) ‘The hard art of soft science’. Journal of Evaluation in Clinical Practice.

[9] Grypdonck MHF (2006). Qualitative health research in the era of evidence–based practice. Qualitative Health Research.

[10] Little M (2003). ‘Better than numbers …’ A gentle critique of evidence-based medicine. ANZ Journal of Surgery.

[11] Saarni SI, Gylling HA (2004). Evidence based medicine guidelines: a solution to rationing or politics disguised as science?. Journal of Medical Ethics.

[12] Sinclair S (2004). Evidence-based medicine: a new ritual in medical teaching. British Medical Bulletin.

[13] Valkenburg G, Achterhuis H, Nijhof A (2003). Fundamental shortcomings of evidence-based medicine. Journal of Health Organization and Management.

[14] Morse JM (2005). Beyond the clinical trial: expanding criteria for evidence. Qualitative Health Research.

[15] Berguer R (2004). The evidence thing. Annuals of Vascular Surgery.

[16] Berwick DM (2008). The science of improvement. Journal of the American Medical Association.

[17] Danzer G, Rose M, Walter M, Klapp BF (2002). On the theory of individual health. Journal of Medical Ethics.

[18] Vandenbroucke JP (1999). Case reports in an evidence-based world. Journal of the Royal Society of Medicine.

[19] Vineis P (2004). Evidence-based medicine and ethics: a practical approach. Journal of Medical Ethics.

[20] Greenhalgh T (1999). Narrative-based medicine in an evidence-based world. British Medical Journal.

[21] Sackett DL, Rosenberg WMC, Gray JAM, Haynes RB, Richardson WS (1996). Evidence-based medicine: what it is and what it isn't. British Medical Journal.

[22] Malterud K (2001). The art and science of clinical knowledge: evidence beyond measures and numbers. Lancet.

[23] Rycroft-Malone J, Seers K, Titchen A, Harvey G, Kitson A, McCormack B (2004). What counts as evidence in evidence-based practice?. Journal of Advanced Nursing.

[24] Walshe CE, Caress AL, Chew-Graham C, Todd CJ (2004). Case studies: a research strategy appropriate for palliative care?. Palliative Medicine.

[25] Ghosh AK (2004). On the challenges of using evidence-based information: the role of clinical uncertainty. Journal of Laboratory and Clinical Medicine.

[26] Buetow S, Kenealy T (2000). Evidence-based medicine: the need for a new definition. Journal of Evaluation in Clinical Practice.

[27] Greenhalgh T, Worrall JG (1997). From EBM to CSM: the evolution of context-sensitive medicine. Journal of Evaluation in Clinical Practice.

[28] Porta M (2004). Is there life after evidence-based medicine?. Journal of Evaluation in Clinical Practice.

[29] Demakis JG, McQueen L, Kizer KW, Feussner JR (2000). Quality enhancement research initiative (QUERI): a collaboration between research and clinical practice. Medical Care.

[30] Muhr T (2004). Atlas.ti Version 5.0.

[31] Patton MQ (1990). How to Use Qualitative Methods in Evaluation.

[32] De Simone J (2006). Beyond ‘faith-based medicine’ and EBM. Journal of Evaluation in Clinical Practice.

[33] Parker LE, Kirchner JE, Bonner LM, Yano EM, Fickel JJ, Ritchie MJ, Simons CE (2009). Creating a quality improvement dialogue: utilizing knowledge from frontline staff, managers, and experts to foster health care quality improvement. Qualitative Health Research.

[34] Parker LE, de Pillis E, Altschuler A, Rubenstein LV, Meredith LS (2007). Balancing participation and expertise: a comparison of locally and centrally managed healthcare quality improvement within primary care practices. Qualitative Health Research.

